# 

**Published:** 1864

**Authors:** 


### CHICAGO MEDICAL EXAMINER.

**N. S. DAVIS, M.D., EDITOR.**

A MONTHLY JOURNAL

DEVOTED TO THE

:DUCATIONAL, SCIENTIFIC, AND PRACTICAL INTERESTS

OF THE

**MEDICAL PROFESSION.**

The EXAMINER will be issued during the first week of each month, com-
encing with January, 1860. Each number will contain 64 pages of reading
atter, the greater part of which will be filled with such contents as will
rectly aid the practitioner in the daily practical duties of his profession.
To secure this object fully, we shall give, in each number, in addition to
dinary original articles,, and selections on practical subjects, a faithful re-
rt of many of the more interesting cases presented at the Hospitals and
llege Cliniques. While aiming, however, to make the EXAMINER eminently
ctical, we shall not neglect either scientific, social, or educational interests,
the profession. It will not be the special organ of any one institution,
iety, or clique; but its columns will be open for well-written articles from
respectable member of the profession, on all topics legitimately within the
nain of medical literature, science, and education.
'erms, $2.00 per annum, invariably in advance.

